# Simvastatin in the Prevention of Recurrent Pancreatitis: Design and Rationale of a Multicenter Triple-Blind Randomized Controlled Trial, the SIMBA Trial

**DOI:** 10.3389/fmed.2020.00494

**Published:** 2021-02-10

**Authors:** Karina Cárdenas-Jaén, Alicia Vaillo-Rocamora, Ángel Gracia, Pramoud K. Garg, Pedro Zapater, Georgios I. Papachristou, Vikesh K. Singh, Bechien U. Wu, Enrique de-Madaria

**Affiliations:** ^1^Pancreatic Unit, Department of Gastroenterology, Alicante University General Hospital, Alicante Institute for Health and Biomedical Research (ISABIAL), Alicante, Spain; ^2^Pharmacy Department, Alicante University General Hospital, Alicante Institute for Health and Biomedical Research (ISABIAL), Alicante, Spain; ^3^Department of Gastroenterology, All India Institute of Medical Sciences, New Delhi, India; ^4^Department of Clinical Pharmacology, Alicante University General Hospital, Alicante Institute for Health and Biomedical Research (ISABIAL), Alicante, Spain; ^5^Division of Gastroenterology, Hepatology and Nutrition, Ohio State University Wexner Medical Center, Columbus, OH, United States; ^6^Pancreatitis Center, Division of Gastroenterology, Johns Hopkins Medical Institutions, Baltimore, MD, United States; ^7^Center for Pancreatic Care, Division of Gastroenterology, Kaiser Permanente Los Angeles Medical Center, Los Angeles, CA, United States

**Keywords:** hydroxymethylglutaryl-CoA reductase inhibitors, statins, simvastatin, acute pancreatitis, chronic pancreatitis, recurrent, idiopathic, prevention

## Abstract

**Background:** One in every four patients with a first episode of non-gallstone-related acute pancreatitis (AP) develops recurrent disease. Recurrent episodes of AP or acute flares of chronic pancreatitis (CP) are associated with decreased quality of life and progression of the disease. Besides removing the etiology of pancreatitis (which sometimes is not possible), there are no effective measures to prevent recurrence. Meta-analyses of randomized controlled trials, as well as epidemiological and cohort studies, suggest that statins may be protective against the development of index AP.

**Methods:** The SIMBA study is a triple-blind randomized placebo-controlled, parallel-group multicenter trial. Patients with recurrent AP or with acute flares of CP (at least two episodes in the last 12 months) will be randomized to receive simvastatin 40 mg daily or placebo. During a 3-year study period, 144 patients (72 per arm of treatment) from 26 centers will be enrolled. The patients will receive the study treatment for 1 year. The primary aim is to compare the recurrence of AP or acute flares in CP. Secondary endpoints include the incidence of new-onset diabetes mellitus, new-onset exocrine pancreatic insufficiency (EPI), new-onset imaging signs of CP, frequency of all-cause hospital admissions, severity of AP, adherence to treatment, and frequency of adverse events.

**Discussion:** The SIMBA trial will ascertain whether simvastatin, a safe, widely used and inexpensive drug, can change the natural course of recurrent pancreatitis.

**Trial Registration**: ClinicalTrials.gov Identifier: NCT04021498

## Introduction

Acute pancreatitis (AP) is the third most common cause of hospital admission due to gastrointestinal disease (1). Gallstones and excessive alcohol intake account for most cases of AP. Recurrent AP (RAP) refers to the development of at least two separate documented episodes of AP with a period of resolution in between (2). Approximately 20% of the patients will relapse after a first episode of AP (2, 3). The approximate incidence of recurrent AP is likely 8–10 per 100,000 per year, and its prevalence is 110–140 per 100,000 populations (2). The relatively low frequency of relapse in biliary AP (close to 10%) (3) is due to the high effectiveness of cholecystectomy (4), but a first episode of AP due to alcoholic or other etiologies is associated with relapse in one in every four patients (2, 3). Currently, besides removing etiological factors (which is frequently not possible), there are no specific medical treatment that changes the natural history of RAP. RAP is an intermediate stage in the pathogenesis of chronic pancreatitis (CP) as a subset of RAP patients' transition to CP (one in every three patients) over their natural history (2, 5). Forty-five percent of patients with CP experience intermittent flares of pain according to a prospective cohort study (6). Intermittent pain in CP is associated with missed days of work, frequent need for hospitalization, and a decreased quality of life (6).

Statins are drugs that inhibit 3-hydroxy-3-methyl-glutaryl-coenzyme A reductase, the rate-controlling enzyme in the cholesterol synthetic pathway, resulting in decreased serum levels of total and low-density lipoprotein cholesterol. Besides this, statins may have anti-inflammatory properties (7). Some studies suggest that statins have an important effect on the incidence and severity of AP (8). A meta-analysis of randomized controlled trials showed that statin use was associated with a decreased risk of AP (9). A retrospective cohort study based on data from an integrated health care system suggested that simvastatin is independently associated with a lower probability of having an episode of AP (adjusted risk ratio 0.626, 95% confidence interval 0.588–0.668) (10). Furthermore, some studies have shown decreased severity of AP among consumers of statins (11, 12). We hypothesized that simvastatin, a widely used patent-free statin, reduces the number of new episodes of AP or inflammatory relapses in CP in patients with recurrent pancreatitis. The main aim of SIMBA (**SIM**vastatin in the prevention of recurrent pancreatitis, a triple **B**lind r**A**ndomized controlled multicenter trial) is to compare the recurrence rate of AP in patients with recurrent pancreatitis consuming simvastatin vs. placebo.

## Patients and Methods

### Design

SIMBA is a triple-blind randomized placebo-controlled, parallel-group, superiority multicenter trial. This final protocol (version 4) was finished on June 6th, 2018. This study protocol follows the SPIRIT guidelines (13).

### Participating Centers

The members of the Spanish Association of Gastroenterology (AEG) and the Spanish Association of Pancreatology (AESPANC) were invited to participate in the study between January 2016 and October 2017. Currently (April 2020), 32 Spanish centers and 1 Indian center have agreed to join or are currently recruiting patients.

### Primary Endpoint

The primary endpoint is recurrence of pancreatitis during the 1-year follow-up period (meaning a new attack of AP or acute flares in CP). The definition of recurrent pancreatitis, both in AP and CP, requires at least two of the following three features: (i) typical acute pancreatic abdominal pain (acute onset of a persistent, severe, epigastric pain often radiating to the back); (ii) serum lipase activity (or amylase activity) at least three times greater than the upper limit of normal; and (iii) characteristic findings of pancreatic inflammation on contrast-enhanced computed tomography (CECT) and less commonly magnetic resonance imaging (MRI) or transabdominal ultrasonography (14). Although this definition was originally intended for AP (14), in SIMBA, it is also used to define acute flares of pain and inflammation in CP. Patients with CP and pain without increased serum pancreatic enzymes and/or signs of new-onset inflammation on imaging are not considered as having an inflammatory flare.

### Secondary Endpoints

- New-onset diabetes at the end of follow-up, according to the American Diabetes Association criteria (15). Blood levels of glycosylated hemoglobin at the end of follow-up will also be compared to baseline.
- New-onset exocrine pancreatic insufficiency (EPI) defined by fecal elastase-1 <100 mcg/g (16). Fecal elastase-1 levels at the end of follow-up will also be compared to baseline.
- Imaging signs of CP at the end of follow-up, defined as calcifications and/or dilated pancreatic duct (≥4 mm) (17), mainly on a CT scan, but endoscopic ultrasound and/or MRI are allowed, particularly in younger patients to avoid excessive exposure to ionizing radiation.
- Frequency of all-cause hospital admissions.
- Severity of AP according to the revision of the Atlanta Classification (moderate-to-severe vs. mild) (14).
- Adherence to treatment (percentage of the planned treatment consumed by the patient).
- Frequency of adverse events.

### Study Population

Patients with recurrent pancreatitis managed in the outpatient setting of the recruiting centers are potential candidate subjects for the study. The acute episode of pancreatitis, or acute flares of pain in CP, may have been treated in other centers; however, patients are eligible for recruitment only when all the required information is available. Patients that meet the inclusion and have no exclusion criteria receive detailed information about the study and are asked for written consent to participate.

#### Inclusion Criteria

1. Adult (≥18) patients.
2. At least two episodes of AP or acute flares of CP, defined according to the Revised Atlanta Classification (14).
3. Written informed consent to participate in the study.

#### Exclusion Criteria

1. Less than two episodes of AP or acute flares of CP in the last 12 months.
2. Statin consumption in the previous year.
3. Contraindications to the use of statins (myopathy, allergy, severe liver disease, and drugs that inhibit CYPP3A4).
4. Cholelithiasis or choledocholithiasis diagnosed in the last episode of AP (every patient must have at least one abdominal ultrasonography and a magnetic resonance cholangiopancreatography and/or endoscopic ultrasonography ruling out cholelithiasis to enter the study).
5. Endoscopic sphincterotomy and/or cholecystectomy and/or pancreatic surgery between last episode of pancreatitis and recruitment or patients who are expected to undergo any of these interventions within the next year.
6. Serum triglycerides >500 mg/dl without previous specific treatment before the last episode of pancreatitis or patients expected to have a change in their specific hypertriglyceridemia treatment in <1 year.
7. Primary hyperparathyroidism that has been operated between last episode of pancreatitis and recruitment or will be operated in <1 year.
8. Iatrogenic AP (pancreatitis due to endoscopic retrograde cholangio-pancreatography, surgery, or after other invasive treatment). Iatrogenic pancreatitis will not count as an episode of recurrent pancreatitis, but patients may be included in the study if they meet the other inclusion and exclusion criteria, specially exclusion criteria number 5.
9. Abstinence syndrome due to alcohol or drugs and/or delirium tremens in the last 6 months before recruitment.
10. Previous (last year) failure to attend follow-up medical visits or lack of adherence to treatment, or social problems that may be associated to lack of adherence to the study treatment or to inadequate follow-up.
11. Pregnancy and breast-feeding.

### Flowchart According to Consort

**Figure 1:**
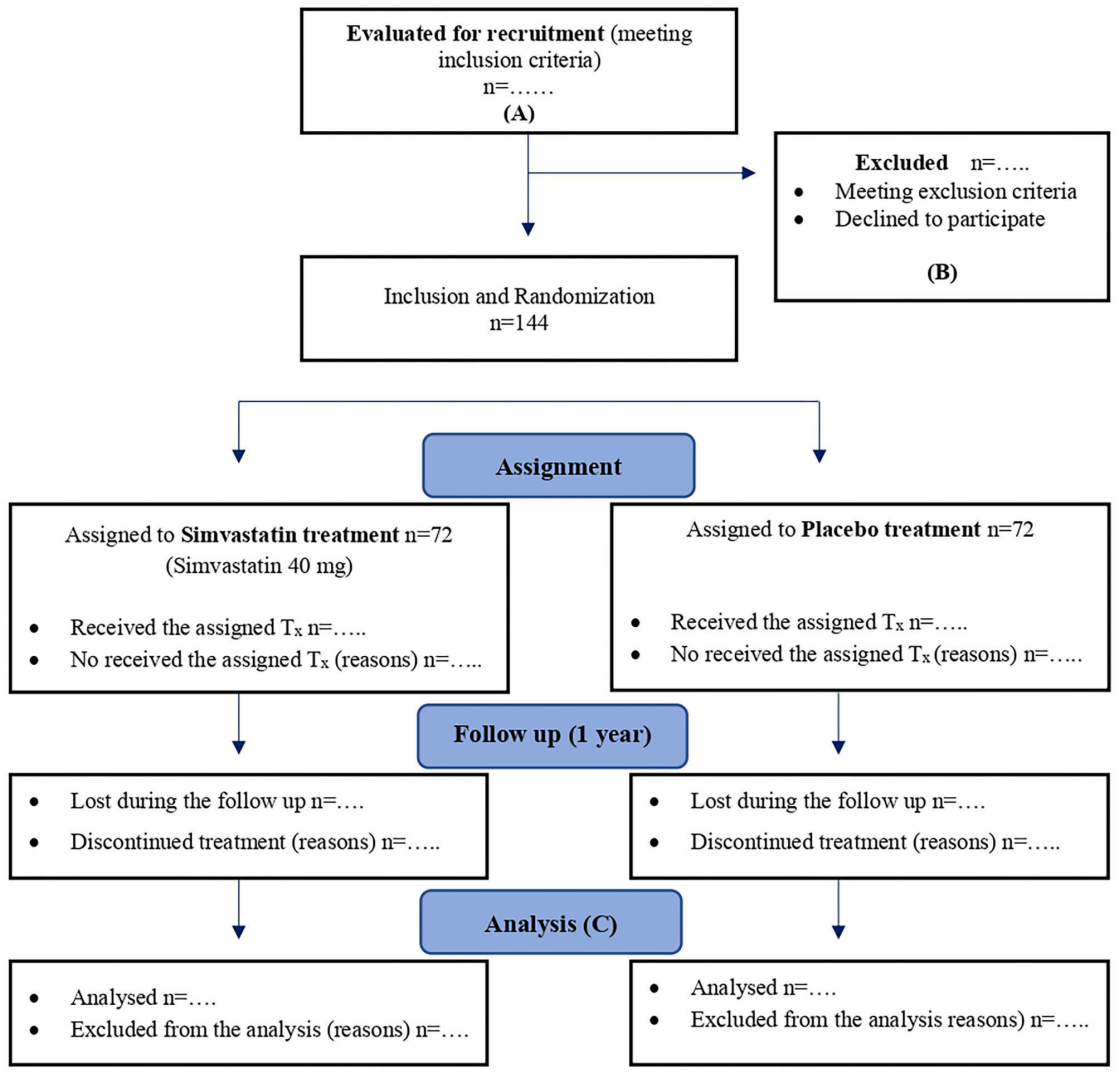
Flowchart for SIMBA trial according to CONSORT 2010 and SPIRIT 2013 recommendations. **(A)** ≥18 years old, at least 2 episodes of AP or acute flares of chronic pancreatitis, written informed consent. **(B)** <2 episodes of pancreatitis or acute flares of chronic pancreatitis in the last 12 months; stain consumption in the previous year; contraindications to the use of statins; cholelithiasis or choledocholithiasis diagnosed in the last episode of pancreatitis; endoscopic sphincterotomy and/or cholecystectomy and/or pancreatic surgery between last episode of AP and recruitment or patients who are expected to undergo one of this techniques in less than a year; serum triglycerides >500 mg/dL without previous specific treatment before the last episode of pancreatitis, or in patients expected to have a change in their specific hypertriglyceridemia treatment in <1 year; primary hyperparathyroidism that has been operated between last episode of pancreatitis and recruitment or will be operated in <1 year; iatrogenic pancreatitis; abstinence syndrome due to alcohol or drugs and/or delirium tremens in the last 6 months before recruitment; previous (last year) failure to attend follow-up medical visits, social problems that may be associated to failure to take the medication or to perform an adequate follow-up; pregnancy or breastfeeding. **(C)** Intention-to-treat (primary analysis) and per-protocol analysis. T_x_, treatment.

The flowchart for the SIMBA trial will be based on the CONSORT recommendations (18, 19) and SPIRIT guidelines (13) (Figure 1).

### Randomization, Masking, and Blinding

Centralized randomization (1:1) is performed by the Clinical Pharmacology Department (PZ) of Alicante's University General Hospital (AUGH). Randomization is based on a computer-generated list (one list per center) of random numbers generated by means of the block-random command from the psych package (20) for R (21). It is used randomization in permuted blocks (each block containing eight patients) stratified by (1) center, (2) more than three lifetime episodes of AP or acute flares of CP, and (3) alcoholic etiology. Only three persons have access to the abovementioned lists: PZ, AVR, and AG, who are in charge of randomization (PZ), coordination of the study (AVR), and masking (AG). None of these individuals will participate in the statistical analysis.

Masking is performed by the Pharmacy Department of AUGH. Both simvastatin and placebo (lactose, excipient of simvastatin) are masked in indistinguishable white capsules. AVR is in charge of checking the randomization lists with the information provided by the study recruiters (assignment of patients to intervention) and sending the medication to the study centers. The final statistical analysis will be performed blindly by EdM (treatment arms will be identified as *label A* and *B*). Only after all statistical analysis is finished will the study arms be unblinded by the Pharmacology Department.

### Treatment Protocol

Patients receive simvastatin 40 mg or placebo, one capsule daily for 1 year. In case of recurrence of AP or acute flares of CP, the patients are advised to continue the treatment as planned. Adherence to treatment is monitored in each outpatient visit (comparison of the number of capsules remaining in the container with the number of capsules that should remain).

According to patient exclusion criteria, endoscopic retrograde cholangio-pancreatography, parathyroid, biliary, and/or pancreatic surgery are discouraged during the treatment period. In case of undergoing any of these procedures, the patient will be included in the intention-to-treat analysis, but the Trial Steering Committee will decide whether that patient should be included in the per-protocol analysis or not (the decision will be taken before blinded statistical analysis).

### Data Management

Clinical data are collected by the study recruiters (gastroenterologists from the participating centers) by means of a standardized electronic case report form (CRF) based on REDCap (22). Access to REDCap is provided by the Spanish Association of Gastroenterology, AEG (AEG REDCap node). The CRF does not contain the name, initials, or any personal identification number from the patients. Each patient is identified in the CRF by a double registration number: center and patient. Only the recruiting gastroenterologists, in direct charge of managing the patient, have access to the patient identity. The CRF has been designed to help promote data quality (including range checks for dates and quantitative data values).

### Recruitment and Follow-Up

Patients are recruited by gastroenterologists specialized in pancreatic disorders in the participating centers. In the initial recruitment visit, the patient receives detailed information about the study; those patients willing to participate must sign the written consent first, and the recruiter proceeds to baseline data collection; the patient will subsequently receive medication or placebo for 1 month. Follow-up outpatient visits take place after 1, 4, 8, and 12 months thereafter. The first visit takes place after just 1 month to monitor for possible early side effects of the medication. Adherence to treatment, alcohol and tobacco consumption, number and severity of new episodes of AP or acute flares of CP, and other causes of hospitalization are registered on each visit, as well as possible secondary effects of the medication. General laboratories are obtained at every outpatient visit. Fecal-elastase 1 and glycosylated hemoglobin are measured at baseline and in the last visit. A CT scan is performed at recruitment and at the end of follow-up. Follow-up visits and tests are performed even in patients who stop taking the study drug, for intention-to-treat analysis.

### Data Monitoring Committee

The role of the Data Monitoring Committee (DMC) is to monitor the data emerging from the trial and to advise the Trial Steering Committee on whether there are any reasons for the trial not to continue (23). The members of the DMC are AVR and PZ.

### Independent Trial Steering Committee

The role of the Trial Steering Committee (TSC) is (1) to monitor and supervise the progress of the trial toward its objectives, (2) to review at regular intervals relevant information from other sources (i.e., other related trials), (3) to consider the recommendations of the DMC, and (4) to advise the Principal Investigator (PI) on the presentation of all aspects of the trial (24). The members of the TSC are BUW, VKS, and GIP.

### Safety

Simvastatin is a widely consumed and safe drug. The collaborating investigators must report immediately any possible adverse reaction. The DMC will be in charge for safety monitoring. In case of a potentially severe adverse reaction, the DMC will consider unblinding that particular patient, if medically advised. The DMC will periodically review the unblinded safety variables. The DMC will contact the Spanish Drug Agency (Agencia Española de Medicamentos y Productos Sanitarios, AEMPS) and the Central Drugs Standard Control Organization (CDSCO) from India, as well as the regional health authorities in case of safety issues.

### Statistical Aspects

For all statistical analysis, the threshold for defining statistical significance will be 0.05.

#### Sample Size Calculation

Based on a pilot retrospective internal non-published analysis of patients from AUGH meeting the study inclusion and exclusion criteria, a recurrence rate of 50% in 1 year in patients with placebo is expected. We powered the study to detect a 50% decrease in recurrence during the study period. According to the arcsine method, with an alpha level of 0.05, a statistical power of 80%, an expected 1-year recurrence in the placebo arm of 50 and 25% in the simvastatin arm, and a 20% loss of follow-up, 144 patients are needed (72 per treatment arm).

#### Descriptive Statistics

Continuous data will be evaluated for normality by the Shapiro–Wilk test and will be summarized using mean and standard deviation or median and interquartile range depending on the variable distribution. Qualitative data will be displayed as *n* (%). Baseline criteria are age, gender, diagnoses of CP, number of episodes of AP or acute flares of CP (lifetime episodes as well as episodes in the 12 months prior to recruitment), etiology, active consumption of alcohol (any alcohol and ≥5 drinks per day) (25), active smoking (25), body mass index, diabetes mellitus (15), and pancreatic exocrine insufficiency (fecal elastase-1 <100 mcg/g).

#### Analysis

For blinded statistical analysis, the Clinical Pharmacology Department of AUGH will provide for every patient included in the study a label: “A” or “B.” After statistical analysis, labels A and B will be unblinded as placebo or simvastatin. Both intention-to-treat (primary analysis) and per-protocol analysis will be performed.

Recurrence of AP or acute flares of CP during the follow-up period will be analyzed as a dichotomous variable (primary analysis and variable used for sample calculation): recurrence during follow-up yes/no, by means of the Chi-square test as well as the Kaplan–Meier time-to-event test, and as a quantitative variable (secondary endpoint): number of episodes of AP or acute flares of CP during follow-up and decrease in the number of episodes in respect to the previous year by means of the *t*-test and pairwise *t*-test, respectively. In case of a non-parametric distribution, the Mann–Whitney *U* test or Wilcoxon test will be performed.

Similarly, for other secondary endpoints, we will use Chi-square test, *t*-test/Mann–Whitney *U* test, or pairwise *t*-test/Wilcoxon test according to the characteristics of the variables. Incidence rate ratio will be used to analyze the reduction in total number of AP events or acute flares of CP per arm of treatment.

Odds ratio (95% confidence interval) will be used for quantifying effect size when applicable. Pre-specified subgroup analysis: patients with AP and patients with CP.

In case of significant difference in the baseline distribution of any variable, multivariate analysis (binary logistic regression) will be used to correct it.

#### Additional Analyses

Subgroup analysis will be performed regarding alcoholic/non-alcoholic etiology. Unplanned additional analysis will be identified in the final article as *post hoc* analysis.

#### Changes in the Protocol and Premature Termination of the Study

In case of slow recruitment rate (<72 patients in 3 years), the TSC may decide an interim analysis. The analysis will only be available to the members of the TSC, who will decide whether the study should continue or not. The DMC will perform regular safety analyses and may ask the TSC for premature termination of the study in case of a safety issue. This scenario is not expected given the wide experience with simvastatin in the last two and a half decades. The TSC may suggest changes to the protocol (for example, in case of very slow recruitment rate or safety issues); in such cases, researchers, drug agencies, and ethics committees will be informed.

### Other Considerations

Enrique de-Madaria will have access to the final trial dataset, but the study arm treatment labels will be blinded until analysis is finished, as explained above. *Post hoc* collateral studies may be performed by the study collaborators with the final database after approval by the sponsor, the TSC, and the central Institutional Review Board.

The results of the study will be reported following the CONSORT Statement (19). The results of the trial will be communicated to patients and researchers, and there will be no publication restrictions. The manuscript draft will be written by EdM and reviewed by the study collaborators.

## Discussion

Some patients suffer from recurrent episodes of AP or acute flares of CP, with their physicians being unable to offer any effective preventive medications. Most of them have alcoholic or idiopathic etiology (26), as this first episode may trigger other risk factors to induce new episodes of AP (sentinel AP event model) (27). Recurrent pancreatitis is a condition associated with great discomfort and decreased quality of life (28). In almost all cases, it is associated with severe pain and requires hospital admission. Patients are afraid to travel, lose days of work and leisure, and often feel desperate about the random nature of pancreatitis flares.

SIMBA aims to investigate whether simvastatin is useful for preventing new episodes of pancreatitis in recurrent AP and CP, avoiding the natural progression of disease (AP) to CP, or the development of exocrine or endocrine pancreatic insufficiency in patients with established CP. We will use fecal elastase to detect EPI; this test is associated to a high false-positive rate (29); for that reason, we choose a 100 mcg/g threshold, which has been suggested to detect severe EPI (16). Based on the studies described in the *Introduction*, we are looking for a new indication for a well-established drug [it was released for medical use in late 80s (30)]. It is an inexpensive drug without active patent; currently, in Spain, treatment with simvastatin 40 mg costs 2.17 euros (2.58 USD) per month. This is a researcher-driven study (we report no conflict of interest) and is financed by public and private grants from the Spanish Government, the Spanish Association of Gastroenterology, and the Alicante's Institute for Health and Biomedical Research (ISABIAL); none of these institutions have commercial interest in the results of the present trial. The PI promoter, the collaborating researchers and TSC members participate in the study in an altruistic manner, without receiving economic compensation. All these considerations, together with the triple-blind design, make SIMBA a solid and clinically relevant study.

The main potential issue will be recruitment rate, as the study criteria are very restrictive; for this reason, we made an important effort for many centers throughout Spain to join. Currently (April 2020), 47 patients have been recruited so far. Another potential issue is adherence to treatment: patients with new episodes of pancreatitis during the follow-up period may abandon the study. For this reason, an estimated 20% loss of follow-up rate was considered in the sample size calculation.

In conclusion, the SIMBA study is a researcher-driven triple-blind randomized placebo-controlled, parallel-group, multicenter trial aiming to compare recurrence of new episodes of pancreatitis in patients with recurrent AP or acute flares of CP, consuming simvastatin vs. placebo.

## Ethics Statement

This study involving human participants was performed in accordance with the declaration of Helsinki as well as the Good Clinical Practice international ethical and scientific quality standards. The study was reviewed and approved by (Comité Ético de Investigación Clínica con Medicamentos del Hospital General Universitario de Alicante, CEIM HGUA, reference number 2016/26). The study was originally approved on July 29th 2016. Secondary approval was obtained from all local Institutional Review Boards. The patients/participants provided their written informed consent to participate in this study.

## Author Contributions

Ed-M is the trial sponsor and PI. The study was designed by Ed-M and BW and initially reviewed by GP, PZ, PG, VS, KC-J, AG, and AV-R. All authors critically assessed the study design, edited the manuscript, and read and approved the final manuscript.

## Collaborators

Deepak Gunjan (Department of Gastroenterology, All India Institute of Medical Sciences, New Delhi, India), María Francisco-González (Department of Gastroenterology, Complexo Hospitalario Universitario de Ourense, Ourense, Spain), Franco Baiocchi-Ureta (Department of Gastroenterology, Complexo Hospitalario Universitario de Ourense, Ourense, Spain), Eduardo Bajador-Andreu (Department of Gastroenterology, Hospital Universitario Miguel Servet, Aragón Health Research Institute (IIS Aragón), Zaragoza, Spain), Federico Bolado (Department of Gastroenterology, Complejo Hospitalario de Navarra, Pamplona, Spain), Carlos Marra-López (Department of Gastroenterology, Complejo Hospitalario de Navarra, Pamplona, Spain), Mar Concepción-Martín (Department of Gastroenterology, Hospital de la Santa Creu i Sant Pau, Institut de Reçerca - IIB Sant Pau, Universitat Autònoma de Barcelona, Barcelona, Spain), Carlos Guarner Argente (Department of Gastroenterology, Hospital de la Santa Creu i Sant Pau, Institut de Reçerca - IIB Sant Pau, Universitat Autònoma de Barcelona, Barcelona, Spain), Andrés J del Pozo-García (Department of Gastroenterology, Hospital 12 de octubre, Madrid, Spain), Adolfo del-Val-Antoñana (Department of Gastroenterology, Hospital La Fe, Valencia, Spain), Robin Rivera-Irigoin (Department of Gastroenterology, Hospital Costa del Sol, Marbella, Spain), Jennifer Hinojosa-Guadix (Department of Gastroenterology, Hospital Costa del Sol, Marbella, Spain), Guillermo García-Rayado (Department of Gastroenterology, Hospital Clínico Universitario Lozano Blesa, Aragón Health Research Institute (IIS Aragón), Zaragoza, Spain), Judith Millastre-Bocos (Department of Gastroenterology, Hospital Clínico Universitario Lozano Blesa, Aragón Health Research Institute (IIS Aragón), Zaragoza, Spain), Carmen Garrido-Durán (Department of Gastroenterology, Hospital Universitario Son Espases, Palma, Spain), Emma Martínez-Moneo (Department of Gastroenterology, Hospital Universitario Cruces, Bilbao, Spain), Cristina Gil García-Ollauri (Department of Gastroenterology, Hospital Universitario Cruces, Bilbao, Spain), Ángel Gracia (Department of Pharmacy, Hospital General Universitario de Alicante, Institute for Health and Biomedical Research (ISABIAL), Alicante, Spain), J. Enrique Domínguez Muñoz (Department of Gastroenterology and Hepatology, Hospital Clínico Universitario Santiago de Compostela. Health Research Institute (IDIS), University Hospital of Santiago de Compostela, Spain), José Lariño-Noia (Department of Gastroenterology and Hepatology, Hospital Clínico Universitario Santiago de Compostela. Health Research Institute (IDIS), University Hospital of Santiago de Compostela, Spain), Rafael Mejuto Fernández (Department of Gastroenterology and Hepatology, Hospital Clínico Universitario Santiago de Compostela. Health Research Institute (IDIS), University Hospital of Santiago de Compostela, Spain), Rosa Martín-Mateos (Department of Gastroenterology, Hospital Universitario Ramón y Cajal, Madrid, Spain), Isabel Pascual-Moreno (Department of Gastroenterology, Hospital Clínico de Valencia, Valencia, Spain), Juan A. Rodríguez-Oballe (Department of Gastroenterology, Hospital Universitari Santa Maria, Lleida, Spain), M. Lourdes Ruiz-Rebollo (Department of Gastroenterology, Hospital Clínico Universitario, Valladolid, Spain), Pedro J. Rosón-Rodríguez (Department of Gastroenterology, Hospital Quirón Salud, Málaga, Spain), Aida Ortega Alonso (Department of Gastroenterology, Hospital Quirón Salud, Málaga, Spain), Francesc Vida-Mombiela (Department of Gastroenterology, Hospital Sant Joan de Deu, Manresa, Spain), Antonio López-Serrano (Department of Gastroenterology, Hospital Universitario Doctor Peset, Valencia, Spain), Eugenia Lauret-Braña (Department of Gastroenterology, Hospital Universitario Central de Asturias, Oviedo, Spain), Manuel A. Jiménez-Moreno (Department of Gastroenterology, Hospital Universitario, Burgos, Spain), Jesús Leal Téllez (Department of Gastroenterology, Hospital Puerta del Mar, Cádiz, Spain), Alba Lira-Aguilar (Consorci Corporació Sanitària Parc Taulí, Sabadell, Spain), Valentí Puig Diví (Consorci Corporació Sanitària Parc Taulí, Sabadell, Spain), Marta Gallach-Montero (Consorci Corporació Sanitària Parc Taulí, Sabadell, Spain), Diego Ledro-Cano (Department of Gastroenterology, Hospital Público La Merced de Osuna, Sevilla, Spain), Blanca Belloc-Barbastro (Department of Gastroenterology, Hospital General San Jorge, Huesca, Spain), Cristina Verdejo Gil (Department of Gastroenterology, Hospital Universitario Fundación Alcorcón, Madrid, Spain), Leticia Betancon Hernández (Department of Gastroenterology, Complejo Hospitalario Universitario Insular Materno Infantil, Palmas de Gran Canaria, Spain), Claudia Virginia Sánchez Marín (Department of Gastroenterology, Hospital Universtario Marqués de Valdecilla, Santander, Spain), Alvaro Teran Lantaron (Department of Gastroenterology, Hospital Universtario Marqués de Valdecilla, Santander, Spain), María Moris Felgueroso (Department of Gastroenterology, Hospital Universtario Marqués de Valdecilla, Santander, Spain), Eduardo Redondo Cerezo (Department of Gastroenterology, Hospital Universitario Virgen de las Nieves, Spain), Juan Gabriel Martínez Cara (Department of Gastroenterology, Hospital Universitario Virgen de las Nieves, Spain), Francisco Valverde López (Department of Gastroenterology, Hospital Universitario Virgen de las Nieves, Spain), Isabel Conde Amiel (Department of Gastroenterology, Hospital General de Valencia, Spain), Cristina Martínez Pascual (Department of Gastroenterology, Hospital Universitario Los Arcos del Mar Menor, San Javier, Spain), Eva Martí Maqués (Department of Gastroenterology, Hospital Universitario Lucus Augusti, Lugo, Spain).

## Conflict of Interest

The authors declare that the research was conducted in the absence of any commercial or financial relationships that could be construed as a potential conflict of interest.

## Abbreviations

AEG: Spanish Association of Gastroenterology
CECT: contrast-enhanced computed tomography
AEMPS: Agencia Española de Medicamentos y Productos Sanitarios (Spanish Drug Agency)
AP: Acute Pancreatitis
AUGH: Alicantes University General Hospital
CEIC HGUA: Comité Ético de Investigación Clínica del Hospital General Universitario de Alicante (AUGH Institutional Review Board)
CP: Chronic Pancreatitis
CRF: Case Report Form
DMC: Data Monitoring Committee
ISABIAL: Alicante's Institute for Health and Biomedical Research
MRI: magnetic resonance imaging
EPI: exocrine pancreatic insufficiency
PI: principal investigator
SIMBA: SIMvastatin in the prevention of recurrent acute pancreatitis, a triple Blind rAndomized controlled multicenter trial
TSC: Trial Steering Committee.
